# Protein-Loosing Entropathy Induced by Unique Combination of CMV and HP in an Immunocompetent Patient

**DOI:** 10.1155/2012/361892

**Published:** 2012-11-08

**Authors:** S. Chen, G. Lalazar, O. Barak, T. Adar, V. Doviner, M. Mizrahi

**Affiliations:** ^1^Internal Medicine A, Liver Unit, Hebrew University-Hadassah Medical Organization, P.O. Box 12000, Jerusalem IL 91120, Israel; ^2^Pathology Institute, Hadassah Ein Kerem Medical Center, Jerusalem, Israel

## Abstract

Protein-losing gastroenteropathies are characterized by an excessive loss of serum proteins into the gastrointestinal tract, resulting in hypoproteinemia (detected as hypoalbuminemia), edema, and, in some cases, pleural and pericardial effusions. Protein-losing gastroenteropathies can be caused by a diverse group of disorders and should be suspected in a patient with hypoproteinemia in whom other causes, such as malnutrition, proteinuria, and impaired liver protein synthesis, have been excluded. In this paper, we present a case of protein-losing enteropathy in a 22-year-old immunocompetent male with a coinfection of CMV and Hp.

## 1. Introduction

Protein-losing gastroenteropathies can be caused by a diverse group of disorders, in which an increase in intestinal leakage of plasma proteins occurs. This leakage can occur via either mucosal injury or increased lymphatic pressure in the gut. Laboratory findings include reduced serum concentrations of albumin, gamma globulins, fibrinogen, transferrin, and ceruloplasmin. The hypoalbuminemia may lead to edema of the lower extremities. 

A variety of benign and malignant conditions can be associated with protein-losing gastroenteropathy, for example, IBD and gastrointestinal malignancies. However, in otherwise healthy patients, the role of CMV in the pathogenesis has been suggested. A gastric biopsy in a few reported cases demonstrated the presence of CMV [1, 2]. However, most of these patients were children who had a typical benign and transient course and required only supportive therapy [3]. Around 90 cases of gastrointestinal involvement were reported in healthy adult patients, the great majority with colonic involvement, among them none had coinfection with Hp [4, 5]. We describe a case of erosive gastritis with significant protein-loss, admitted to our department for evaluation because of vomiting and abdominal pain. Gastric-mucosal biopsy revealed morphological evidence of both CMV and Hp infection.

## 2. Case Report

A previously healthy, 22-year-old male presented to the emergency room with epigastric pain and vomiting during the week prior to his admission. On medical review, the patient denied fever or diarrhea, he also denied any medication or drug using. Physical examination was significant for slight epigastric tenderness, with no signs of peritonitis. No edema or other findings compatible with fluid retention were noted.

On admission laboratory, results showed marked hypoalbuminemia with an albumin of 21 g/L and total protein of 43 g/L. Additional tests showed Sodium 129 mmol/L (135–145 mmol/L), potassium 4.3 mmol/L (3.5–5 mmol/L), alkaline phosphatase (ALP) 26 U/L (40 to 130 U/L), alanine transaminase (ALT) 271 U/L (6 to 53 U/L), aspartate aminotransferase (AST) 183 U/L (2 to 60 U/L), bilirubin 19 *μ*mol/L (0–17 micromol/L), and Lactate dehydrogenase (LDH) 524 (300–620 U/L). No hyperlipidemia with a cholesterol of 2.6 mmol/L (normal 3–5 mmol/L), HDL 0.5 mmol/L (>0.91 mmol/L), LDL 1.51 mmol/L (0–3.4 mmol/L), or TG 1.3 mmol/L (0–2.3 mmol/L). Mild leukocytosis 11.1 K/*μ*L (4–10 K/*μ*L), with elevated hemoglobin levels 17.4 G/% (12–14 G/%) and a normal platelet count of 30.4 K/*μ*L (14–40 K/*μ*L). Diastase 50 U/L (20–100 U/L), uric acid 266 micro mol/L (150–380 *μ*mol/L), PT-INR 1.33, urine toxic screen, and blood alcohol levels were also negative. Abdominal ultrasound showed normal kidneys with a normal spleen, no ascites, and no evidence of hepatic, biliary, or pancreatic disease. Doppler was normal in the hepatic artery and hepatic and portal veins. Urinalysis was negative for protein. A 24 hr urine collection showed 0.1 G/24 H of total protein (normal range 0–0.25 G/24 H). 

Due to a rapid decline in the albumin level (down to 12 g/L), and elevated liver function tests with no evidence for proteinuria or significant synthetic liver function disturbance further work up was performed. Peripheral smear was normal and a contrast-enhanced total body CT was negative for lymphoadenopathy or mass lesion, with a normal spleen and liver, no ascites, and no evidence of ileal or colonic disease immune serology: antinuclear antibody (ANA): negative, rheumatoid factor (RF): negative, C3: 106 (80–160 m/dL), and C4: 17.4 (15–35 mg/dL). Serologies for hepatitis (A,B,C) were negative, Hunan immunodeficiency virus (HIV) was negative, Epstein Barr virus nuclear antigen (EBNA) was positive, cytomegalovirus (CMV)-IgG Ab was positive 8 IU/ML (negative < 4), and CMV-IgM Ab was positive 1.39 (pos > 0.9), CMV-PCR 1015 copies/mL.

**Figure 1:**
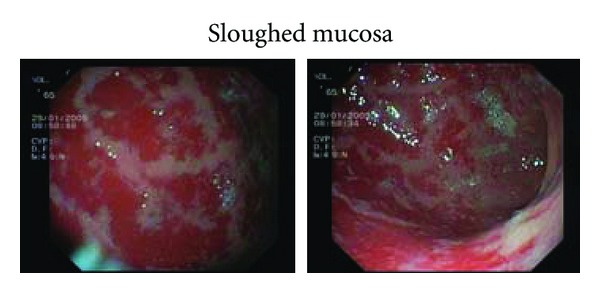
Diffuse severe erosive gastritis with sloughing of the gastric mucosa (antral sparing).

**Figure 2:**
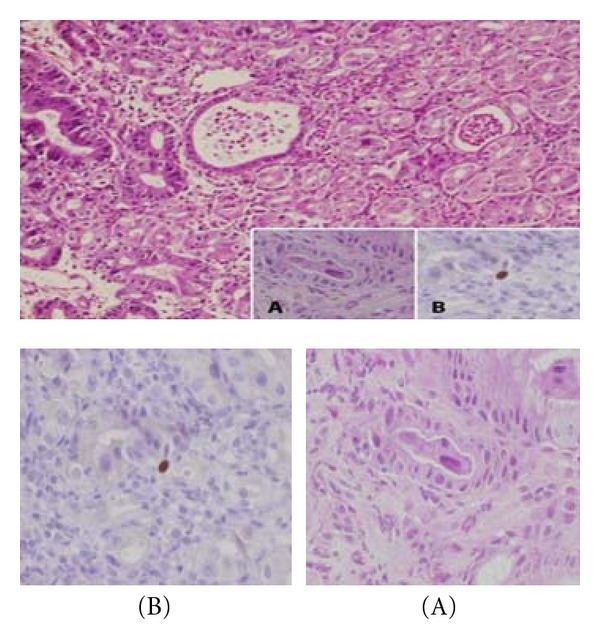
Acute gastritis with pit abscesses. Several intranuclear inclusions were seen in gastric-epithelial cells (A) and were found positive for CMV by immunohistochemistry (B) (H&E, original magnification ×100, insets (A, B) ×400).

Because there was no overt explanation for the extreme hypoalbuminemia, upper GI endoscopy and *α*-1 antitripsin levels were performed to explore the possibility of protein loosing enteropathy. *α*-1 antitripsin levels were in normal range; on endoscopy severe diffuse erosive gastritis with sloughing of the gastric mucosa, and relative antral sparing was demonstrated Figure 1. Rapid urease test was positive. Pathological examination of mucosal biopsies revealed acute gastritis with numerous culvilinear rods proved as *H. pylori* by immunohistochemistry (not shown) and a few viral intranuclear inclusion bodies suggestive of CMV infection and confirmed as such by immunohistochemical stain Figures 2(A) and 2(B). 

 Due to the severe clinical presentation with severe gastritis, profound hypoalbuminemia, and elevated liver enzymes that in our patient was considered to be more as epiphenomena of the clinical picture than involvement of the liver by the MCV infection itself, treatment with intravenous Ganciclovir 5 mg/kg was initiated in combination with triple therapy for Hp (Clarithromycin + Amoxicillin + PPI for 2 weeks). Following this treatment the abdominal pain resolved, enteral feeding was reinstated, albumin levels rose to 32 GR/L (normal range 35–50 GR/L), and liver enzymes went back to normal ALP 28 U/L (40 to 130 U/L), ALT 46 U/L (6 to 53 U/L), AST 51 U/L (2 to 60 U/L), bilirubin 8 *μ*mol/L (0–17 micromol/L), and LDH 470 (300–620 U/L). Following one week of combined therapy, repeat blood tests showed a CMV-IgG Ab level of 15 AU/ML (negative < 4), a CMV-IgM Ab of 1.15 AU/ML (pos > 0.9), and CMV-PCR 180 copies/mL. A breath test to detect HP after one month from treatment was negative.

## 3. Discussion

The wide spectrum of disease caused by the cytomegalovirus (CMV) is largely dependent on the host immune status. CMV infections in immunocompromised patients can cause considerable morbidity and mortality, especially among those infected with the human immunodeficiency virus (HIV) and transplant recipients [6–8]. Infection in the immunocompetent host is usually asymptomatic or may cause the infectious mononucleosis syndrome. However, infrequently, primary CMV infection in an immunocompetent host can lead to severe complications with significant morbidity and mortality [9–11]. Both diseases restricted to a single organ and fulminant, multisystem disorders have been described. However, these cases are uncommon and limited to small series and case reports [8–10]. Gastrointestinal involvement with CMV is uncommon in immunocompetent hosts, but may be associated with a high rate of morbidity and mortality if misdiagnosed [12, 13]. Involvement of the lower GI tract is more frequent than involvement of the upper GI tract. Although CMV colitis in immunosuppressed patients is usually due to reactivation of latent infection in immunocompetent hosts, it can occur also in the setting of primary infection [14, 15]. The most frequent lesion of the GI tract due to CMV is ulceration, which can involve the mucosa of the GI tract from the esophagus to the rectum [16, 17]. The mechanism of injury seen during CMV infection is still controversial, and proposed mechanisms include endothelial ischemic injury due to invasion of endothelial cell by the virus or increased vascular permeability leading to protein loosing enteropathy, which is seen more frequently in children than in adults [18, 19]. CMV infection causing erosive gastritis is a rare condition [20, 21]; recently, involvement of CMV in an acute gastric mucosal lesion (AGML) was described [22, 23]. In this report, the authors concluded that in an AGML CMV, infection should be ruled out using serology, polymerase chain reaction (PCR) [24], and biopsies from the gastric mucosa. 

Helicobacter pylori is a commensal pathogen causing commonly associated with gastric inflammation, erosive gastritis, and gastric ulcers [25, 26]. This pathogen can occasionally cause protein losing gastropathy, but the reports of this association have been rare [27, 28]. To our knowledge, this is the first reported case of protein-losing gastropathy due to the combination of CMV and HP infections in an immune competent adult. 

## 4. Conclusion 

In conclusion, in adult immunocompetent patients, primary CMV infection can involve the gastric mucosa, causing severe gastritis, which may lead to protein loosing gastroenteropathy. Thus, gastric involvement of CMV may be considered in an immunocompetent adult with epigastric pain, severe hypoalbuminemia, and hepatitis. Prompt esophago-gastro-duodenoscopy can facilitate diagnosis and appropriate therapy.

## Abbreviations

CMV: Cytomegalovirus
Hp: Helicobacter pylori
IBD: Inflammatory bowel disease.
